# Predictive Factors of Acute Appendicitis and Its Outcomes Among the Pediatric Age Group

**DOI:** 10.7759/cureus.77925

**Published:** 2025-01-24

**Authors:** Hussain A Al Ghadeer, Abdullah F Al Muaibid, Mohammed A Alkhalaf, Nazihah A Al Nowaiser, Ali A Alkhalaf, Nada N Alghuwainem, Norah N Alharbi, Ahmed M Albuali, Sarah S Almuslim, Noarah A Aljumaiah, Abdulaziz M Alothman, Mohammed I Alhanfoush, Sarah W Albahar, Mariya A Budris, Israa A Alhawas

**Affiliations:** 1 Pediatrics, Maternity and Children Hospital, Al-Mubarraz, SAU; 2 Pediatrics, King Faisal University, Al-Hofuf, SAU; 3 Pediatrics, Vision Colleges, Riyadh, SAU; 4 Pediatrics, Dubai Medical College for Girls, Dubai, ARE

**Keywords:** al ahsa, appendectomy, appendicitis, children, pediatric appendicitis score, saudi arabia

## Abstract

Background

Acute appendicitis in preschool children remains a diagnostic challenge despite advanced imaging techniques’ widespread availability. The majority of these children come late, often with complications such as appendicular perforation, abscess development, and peritonitis. As a consequence, hospital stays are lengthy and linked with an increase in morbidity and mortality. In this research, we aim to predict the factors of acute appendicitis and its outcomes among the pediatric age group.

Methods

We conducted a retrospective study at the Maternity and Children’s Hospital, Al Ahsa, Saudi Arabia, from 2022 to 2023 by reviewing the medical records of pediatric patients younger than 14 years admitted to the ER with acute appendicitis. We divided the patients into either complicated or simple appendicitis. We compared the two groups in terms of baseline characteristics, symptoms, and signs by using the Pediatric Appendicitis Score, duration of symptoms, and length of hospital stay as factors, and we assessed the significant predictive factors for the diagnosis of the type of appendicitis and length of hospital stay.

Results

During the study period, 246 children with a mean age of 10.1 ± 2.2 years and a male predominance of 171 (69.5%) presented with appendicitis. Simple appendicitis affected half of the participants (137, 55.7%) compared to complicated (76, 30.9%). Thirty-three children (13.4%) had a normal appendix. Complex appendicitis affected 76 (30.9%) of cases. Of those who received conservative treatment, 105 (42.7%) had a failure rate of 32 (30.5%). The mean hospital stay was 5.5 ± 4.0 days. Children under five years with complicated appendicitis had high appendicitis scores (p = < 0.05).

Conclusion

The predictive factors for pediatric appendicitis diagnosis are helpful in identifying those children who require intervention. However, the most crucial diagnostic instruments for determining the diagnosis of appendicitis in children are still the clinical signs and a physical abdominal examination.

## Introduction

Acute abdominal pain in children may have a wide variety of causes, including infectious, inflammatory, musculoskeletal, traumatic, gynecologic, and other etiologies. Appendicitis is the most frequent surgical emergency that occurs in children, often caused by a blockage of the lumen with nonspecific causes [1,2]. The risk is 7% in childhood and decreases with age due to lymphoid tissue and vascularity atrophy [3]. Appendicitis is most common in the second decade of life, with a preponderance in boys [4]. On average, one child was diagnosed with appendicitis in 15 children with acute abdominal pain, and one-third of children were diagnosed with perforated appendicitis that ruptured prior to getting treatment [5].

Acute appendicitis is classified into two types: simple appendicitis (early, inflamed, and without complications) and severe appendicitis (gangrenous, perforated appendicitis with abscess/phlegmon, or perforated appendicitis without abscess/phlegmon) [6,7]. Appendicitis is difficult to diagnose in children owing to aberrant presentation, nonspecific symptoms, and a large variety of alternative diagnoses. The initial misdiagnosis rate of appendicitis in older children ranges from 28% to 57% [8]. The rate of perforated appendicitis in pediatric patients younger than three years was as high as 80%-100%, compared to 38% in older children [9,10]. A delayed diagnosis raises the likelihood of complications from appendicitis as well as the risk of morbidity, death, prolonged hospitalization, and increased healthcare costs.

In diagnosing acute appendicitis, laboratory tests play a crucial role. In addition to tests such as white blood cell (WBC) count with differential serum C-reactive protein (CRP), a number of clinical scores, including the pediatric appendicitis score, the Alvarado score, an inflammatory appendix provocative reaction, and a risk calculator for pediatric appendicitis, have been developed to support the diagnostic process. The severity of the abdominal pain, presence of vomiting, expanded internal temperature, WBC count, and CRP level are just a few of the clinical parameters that determine these scores [11-14]. Because these modalities’ sensitivity and explicitness values are insufficient to be used as pure diagnostic tools, they are more helpful for risk separation than for a definitive diagnosis [15,16].

## Materials and methods

In this descriptive study, we retrospectively reviewed 246 pediatric cases with acute appendicitis diagnosed at the Maternity and Children Hospital in Al Ahsa, Saudi Arabia, between January 2022 and December 2023. To evaluate the pattern and outcome of appendicitis, we analyzed the Pediatric Appendicitis Score (PAS), histology findings, and postoperative length of hospital stay. We included patients 14 years old and younger who were admitted to the ER with appendicitis.

In pediatric acute appendicitis, we evaluated the associations between the pathological progression and disease severity and the PAS. We considered gangrenous appendicitis, perforated appendicitis, or the discovery of an abscess formation during surgery as forms of complicated appendicitis. We defined appendicitis other than that previously mentioned as simple appendicitis. We calculated the PAS based on the following parameters (Table 1).

**Table 1:** Pediatric Appendicitis Score (PAS) [17] Risk classification score: low-risk score, <4; moderate risk score, 4-6; high-risk score, >6

Factors	Points
Cough/percussion/hopping tenderness	2
Tenderness in the right lower quadrant	2
Migration of pain	1
Nausea/vomiting	1
Anorexia	1
Fever	1
Leukocyte count ≥ 10 000/lL	1
Polymorphonuclear neutrophilia: neutrophil ≥ 75%	1

Data collection

We collected data from the medical records data registry at the Maternity and Children Hospital. The data covered baseline characteristics, medical history, physical examination findings, duration of symptoms, laboratory and imaging results, final diagnosis, treatment, and length of hospital stay. We divided the patients into normal appendicitis, complicated appendicitis, and noncomplicated appendicitis. We defined appendicitis by radiological and pathological findings obtained from surgery.

Statistical analysis

We collected, reviewed, and then fed the data into IBM SPSS Statistics for Windows, Version 26 (Released 2019; IBM Corp., Armonk, New York, United States). All statistical methods used were two-tailed with an alpha level of 0.05, which we considered significant if the p-value was less than or equal to 0.05. We conducted descriptive analysis for categorical data using frequencies and percentages for children’s demographic data, appendicitis clinical data and pattern, management methods, and clinical outcomes including treatment failure and hospital stay. We graphed radiological findings (ultrasound and CT scan). We used cross-tabulation to show factors associated with pediatric appendicitis severity among children with acute appendicitis and the association between pediatric appendicitis severity and the clinical outcome using Pearson’s chi-squared test and exact probability test for small frequency distributions.

## Results

We reviewed a total of 246 children with acute appendicitis (Table 2). Their ages ranged from one year to 14 years with a mean age of 10.1 ± 2.2 years. A total of 171 (69.5%) were boys, with only 14 (5.7%) known to have a chronic disease. The most reported comorbidity was asthma (6, 42.9%), sickle cell disease (3, 21.4%), Crohn’s disease (1, 7.1%), and others. The children’s weight ranged from 8 kg to 136 kg with a mean weight of 33.4 ± 14.3 kg.

**Table 2:** Biodemographic data of the study children with acute appendicitis (n = 246)

Biodemographic data	No	%
Age in years		
<5 years	6	2.4%
5-9 years	105	42.7%
9-12 years	38	15.4%
>12 years	97	39.4%
Gender		
Male	171	69.5%
Female	75	30.5%
Known to have a chronic disease		
Yes	14	5.7%
No	232	94.3%
If yes, which comorbidity		
Asthma	6	42.9%
Sickle cell disease (SCD)	3	21.4%
Crohn's disease	1	7.1%
Diabetes mellitus (DM)	1	7.1%
Epilepsy	1	7.1%
Glucose-6-phosphate dehydrogenase deficiency (G6PDD)	1	7.1%
Right ureteropelvic junction obstruction	1	7.1%
Weight in Kg		
Range	8-136	
Mean ± SD	33.4 ± 14.3	
Median	31	

Table 3 shows the clinical data and patterns of appendicitis among the pediatric age group in Al Ahsa, Saudi Arabia. As for clinical manifestations, 87 children (35.4%) had low-risk appendicitis, 80 (32.5%) had moderate-risk appendicitis, and 79 (32.1%) had high-risk appendicitis. Symptoms lasted for more than 24 hours in most cases (156, 63.4%). As for the appendicitis type, the most reported was simple appendicitis (137, 55.7%), but 76 cases (30.9%) had complicated appendicitis, and 33 (13.4%) showed a normal appendix. The diameter of the appendix ranged from 0.7 to 109 mm with a median diameter of 8.0 mm.

**Table 3:** Clinical data and patterns of appendicitis among the pediatric age group

Clinical data	No	%
Pediatric Appendicitis Score (PAS)		
Low risk (<4)	87	35.4%
Moderate risk (4-6)	80	32.5%
High risk (>6)	79	32.1%
Duration of symptoms		
Less than 24 hours	90	36.6%
More than 24 hours	156	63.4%
Type of appendicitis		
Simple appendicitis	137	55.7%
Complicated appendicitis	76	30.9%
Normal appendix	33	13.4%
Diameter of appendix (mm)		
Range	0.7-10.9	
Mean ± SD	9.9 ± 10.6	
Median	8.0	

**Figure 1:**
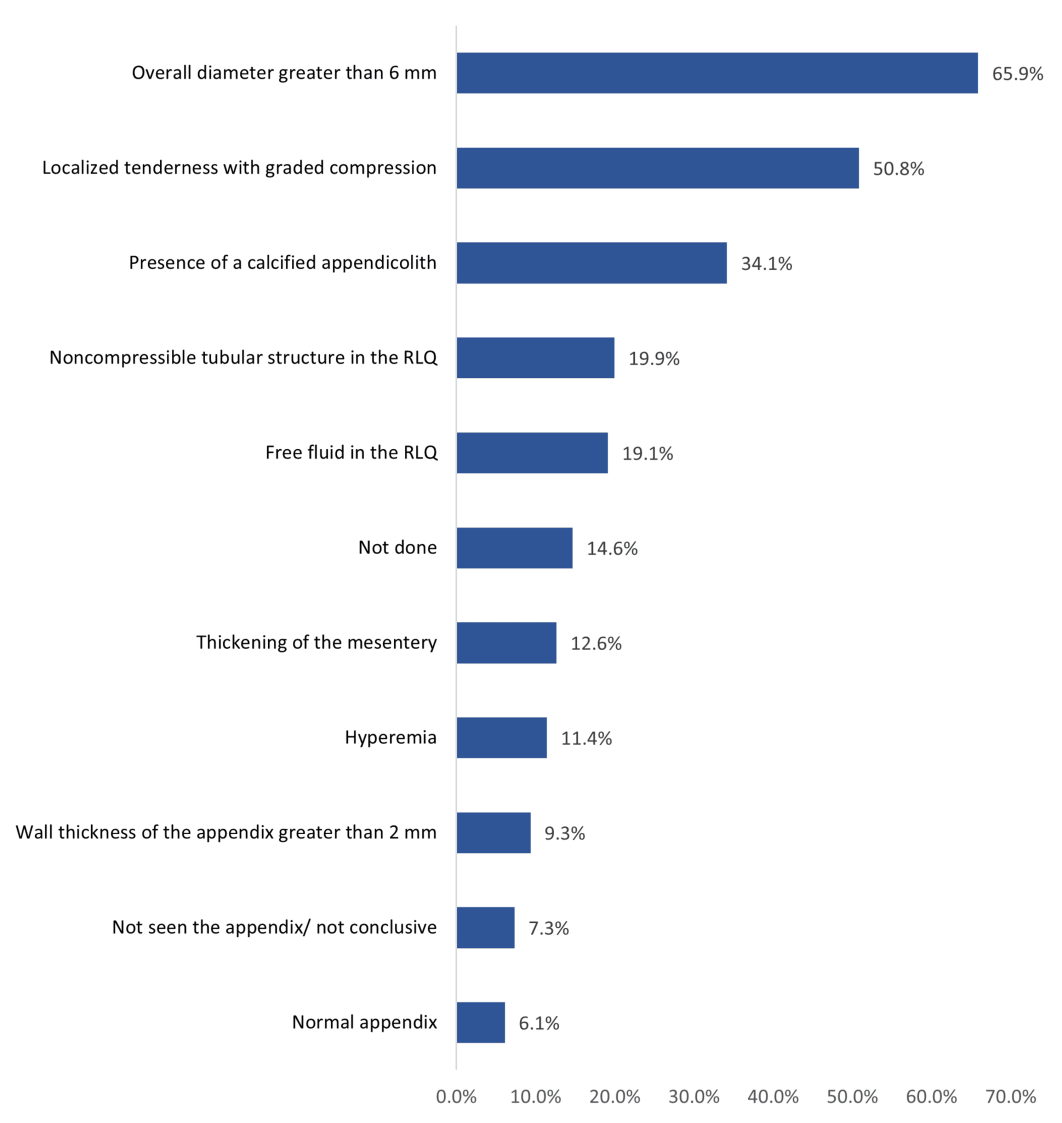
Sonographic findings among children with acute appendicitis RLQ: right lower quadrant

Figure 1 shows the sonographic findings among children with acute appendicitis. The most detected ultrasound findings were overall diameter greater than 6 mm in 162 children (65.9%), localized tenderness with graded compression (125, 50.8%), the presence of a calcified appendicolith (34.1%), noncompressible tubular structure in the right lower quadrant (RLQ) (49, 19.9%), and free fluid in the RLQ (47, 19.1%). A total of 36 children (14.6%) did not have an ultrasound.

**Figure 2:**
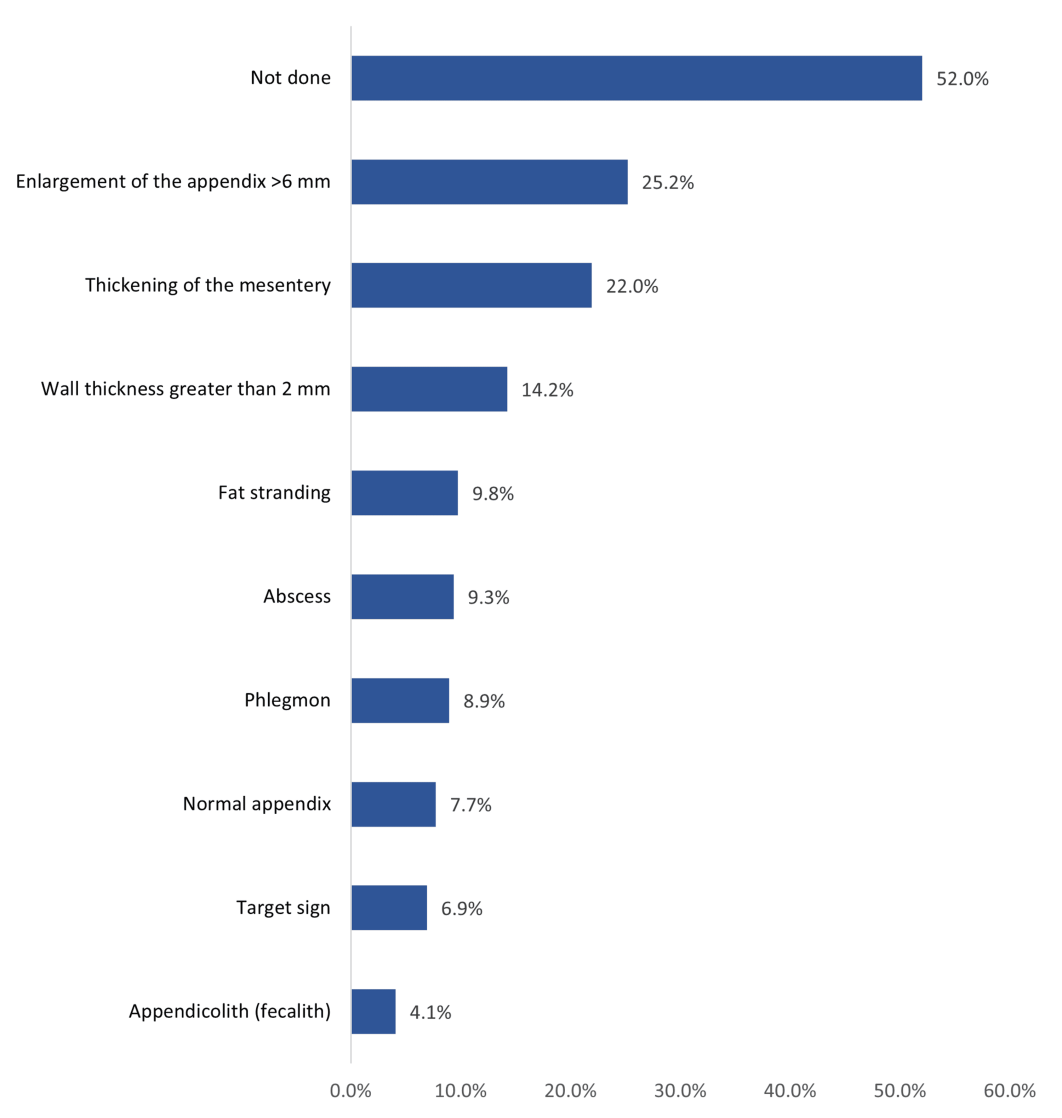
CT scan findings among children with acute appendicitis

Figure 2 shows the CT scan findings among children with acute appendicitis. The most reported findings included enlargement of the appendix (>6 mm) in 62 children (25.2%), thickening of the mesentery (54, 22%), wall thickness > 2 mm (35, 14.2%), fat stranding (24, 9.8%), and abscess (23, 9.3%). Of these children, 128 (52%) did not have a CT scan.

Table 4 presents the treatment and outcome of acute appendicitis among children with acute appendicitis. Of the children, 105 (42.7%) received conservative treatment, 60 (24.4%) underwent a laparoscopic appendectomy, and 81 (32.9%) had an open appendectomy. Thirty-two (30.5%) showed a failure of conservative management, ranging from four to 660 days with a median of 45 days. Fifteen (46.9%) had complicated appendicitis after the failure of treatment. The length of hospital stay ranged from one to 25 days with a mean duration of 5.5 ± 4.0 days.

**Table 4:** Treatment and outcome of acute appendicitis among children with acute appendicitis

Treatment and outcome	No	%
Type of treatment		
Conservative	105	42.7%
Laparoscopic appendectomy	60	24.4%
Open appendectomy	81	32.9%
Failure of conservative management (n = 105)		
Yes	32	30.5%
No	73	69.5%
Type of appendicitis after the failure of conservative management (n = 32)		
Complicated	15	46.9%
Simple appendicitis	17	53.1%
Duration till conservative treatment failure (days)		
Range	4-660	
Mean ± SD	102 ± 143	
Median	45	
Length of stay (days)		
1-3 days	107	43.5%
4-7 days	88	35.8%
8-10 days	30	12.2%
>10 days	21	8.5%
Mean SD	5.5 ± 4.0	

Table 5 presents the factors associated with pediatric appendicitis severity among children with acute appendicitis. Three children (50%) younger than five years had high-risk appendicitis versus seven (18.4%) of those aged 9-12 years, with a recorded statistical significance (p = 0.016). Forty-eight children (63.2%) with complicated appendicitis had high-risk appendicitis versus three (9.1%) who had a normal appendix (p = 0.001). In addition, 15 (65.2%) with an abscess (CT finding) had high-risk appendicitis, 12 (50%) had fat stranding, 11 (50%) had phlegmon, and eight (47.1%) had the target sign compared to four (21.1%) with a normal appendix (p = 0.009). Likewise, 15 (65.2%) of the cases with wall thickness of the appendix had high-severity appendicitis, 40 (47.6%) showed the presence of a calcified appendicolith, and 22 (44.9%) with noncompressible tubular structure in the RLQ had high-risk appendicitis compared to three (20%) with a normal appendix.

**Table 5:** Factors associated with pediatric appendicitis severity among children with acute appendicitis P: Pearson X^2 ^test; ^: exact probability test; *p < 0.05 (significant); RLQ: right lower quadrant

Factors	Pediatric appendicitis severity						p-value
	Low risk (<4)		Moderate risk (4-6)		High risk (>6)		
	No	%	No	%	No	%	
Age in years							.016*^
<5 years	1	16.7%	2	33.3%	3	50.0%	
5-9 years	35	33.3%	28	26.7%	42	40.0%	
9-12 years	10	26.3%	21	55.3%	7	18.4%	
>12 years	41	42.3%	29	29.9%	27	27.8%	
Gender							.248
Male	64	37.4%	50	29.2%	57	33.3%	
Female	23	30.7%	30	40.0%	22	29.3%	
Type of appendicitis							.001*
Complicated appendicitis	9	11.8%	19	25.0%	48	63.2%	
Normal appendix	24	72.7%	6	18.2%	3	9.1%	
Simple appendicitis	54	39.4%	55	40.1%	28	20.4%	
Duration of symptoms							.507
Less than 24 hours	35	38.9%	30	33.3%	25	27.8%	
More than 24 hours	52	33.3%	50	32.1%	54	34.6%	
CT scan finding							.009*
Normal appendix	11	57.9%	4	21.1%	4	21.1%	
Thickening of the mesentery	19	35.2%	19	35.2%	16	29.6%	
Enlargement of the appendix >6 mm	18	29.0%	22	35.5%	22	35.5%	
Wall thickness greater than 2 mm	11	31.4%	14	40.0%	10	28.6%	
Abscess	2	8.7%	6	26.1%	15	65.2%	
Fat stranding	5	20.8%	7	29.2%	12	50.0%	
Phlegmon	5	22.7%	6	27.3%	11	50.0%	
Target sign	3	17.6%	6	35.3%	8	47.1%	
Appendicolith (fecalith)	4	40.0%	2	20.0%	4	40.0%	
Not done	50	39.1%	45	35.2%	33	25.8%	
Sonographic findings							.001*^
Normal appendix	7	46.7%	5	33.3%	3	20.0%	
Overall diameter greater than 6 mm	54	33.3%	48	29.6%	60	37.0%	
Noncompressible tubular structure in the RLQ	8	16.3%	19	38.8%	22	44.9%	
Localized tenderness with graded compression	41	32.8%	31	24.8%	53	42.4%	
Presence of a calcified appendicolith	26	31.0%	18	21.4%	40	47.6%	
Thickening of the mesentery	15	48.4%	10	32.3%	6	19.4%	
Hyperemia	16	57.1%	8	28.6%	4	14.3%	
Not seen the appendix/not conclusive	5	27.8%	7	38.9%	6	33.3%	
Free fluid in the RLQ	15	31.9%	19	40.4%	13	27.7%	
Wall thickness of the appendix greater than 2 mm	5	21.7%	3	13.0%	15	65.2%	
Not done	8	22.2%	18	50.0%	10	27.8%	

Table 6 presents the association between pediatric appendicitis severity and the clinical outcome. Forty-five (57%) children with severe-risk appendicitis underwent an open appendectomy compared to seven (8%) with low-risk appendicitis, while 69 (79.3%) with low-risk appendicitis had conservative treatment (p = 0.001). In addition, 15 (19%) with severe-risk appendicitis stayed in hospital for more than 10 days versus two (2.3%) with low-risk appendicitis, whereas 61 (70.1%) of those with low-risk appendicitis stayed for 1-3 days (p = 0.001).

**Table 6:** Association between pediatric appendicitis severity and the clinical outcome P: Pearson X^2^ test; ^: exact probability test; *p < 0.05 (significant)

Outcome	Pediatric appendicitis score						p-value
	Low risk (<4)		Moderate risk (4-6)		High risk (>6)		
	No	%	No	%	No	%	
Type of treatment							.001*
Conservative	69	79.3%	21	26.3%	15	19.0%	
Laparoscopic appendectomy	11	12.6%	30	37.5%	19	24.1%	
Open appendectomy	7	8.0%	29	36.3%	45	57.0%	
Failure of conservative management							.631
Yes	22	31.9%	7	33.3%	3	20.0%	
No	47	68.1%	14	66.7%	12	80.0%	
Length of stay (days)							.001*^
1-3 days	61	70.1%	26	32.5%	20	25.3%	
4-7 days	20	23.0%	39	48.8%	29	36.7%	
8-10 days	4	4.6%	11	13.8%	15	19.0%	
>10 days	2	2.3%	4	5.0%	15	19.0%	

## Discussion

In this study, we aimed to assess the diagnostic significance of leukocyte count and neutrophil percentage in diagnosing and predicting uncomplicated and complicated appendicitis, respectively. Appendicitis is a surgical emergency and one of the most common causes of abdominal pain, especially in children [18]. It is important to consider it in any patient experiencing sudden abdominal pain who has never had an appendectomy. It is crucial to diagnose it quickly because delaying diagnosis increases the risk of rupture [19-21].

Our study revealed that moat appendicitis children were boys aged 10 years or older. As for the clinical pattern of appendicitis, there was a uniform distribution for the severity score, where nearly one-third had low-risk, one-third had moderate-risk, and one-third had severe-risk appendicitis. Most cases had simple appendicitis with symptoms lasting for more than 24 hours, but less than one-third had complicated appendicitis. Becker et al. [22] found that the average age of pediatric appendicitis cases was 11.9 years old, which is similar to our findings. Likewise, Pearl et al. [23] found that the appendicitis patients’ median age was 12 years (ranging from six months to 18 years), of which 59% were boys. Noh et al. [24] reported that 61% of patients were boys and 39% were girls. As for age, the literature showed that appendicitis cases are often observed in patients aged six to 12, particularly in boys over 10 years old, which is comparable with our study findings [25-27].

In England, Aarabi et al. [28] found that appendicitis was more common in male children than in females. Also, they reported that in most cases, they observed the following signs: absence of fever (83%), absence of Rovsing’s sign (68%), normal or increased bowel sounds (64%), absence of rebound pain (52%), lack of pain migration (50%), lack of guarding (47%), sudden onset of pain (45%), lack of appetite loss (40%), absence of severe pain in the lower right abdomen (32%), and absence of tenderness when the abdomen is tapped (31%), which are all consistent with low-severity cases. In addition, Pearl et al. [23] found that most cases had nonperforated appendicitis (68%) and perforated appendicitis in 279 cases (20%).

Our study also revealed that young age, complicated appendicitis, and radiological signs of wall thickness or severe inflammation (abscess) were associated with a higher severity score. Pearl et al. [23] reported similar findings, where age ≤ eight years was predictive (p < 0.001) of a higher rate of perforated appendicitis. Arslan et al. [29] documented that appendicitis was most frequently observed in the spring and winter seasons. They also found it to be more common in boys aged 10-13 years. Additionally, the frequency of perforated appendicitis increases as age decreases, which is consistent with our findings. The frequency of perforated appendicitis in all age groups was 44% in Gunsar et al.’s study [30]. Yildiz et al. reported that the rate of perforated appendicitis was 20% [31]. In Saudi Arabia, Alnuaymah et al. [32] found that more than half of the cases had acute appendicitis, 36.9% had complicated appendicitis, and 38 cases (1.3%) had no appendicitis histologically.

As for radiological findings, we found that most cases showed a thickness wall, appendicular mass, or dilated appendix diameter with an ultrasound or CT scan. Ultrasonography of the abdomen and pelvis is often performed in the diagnostic workup for acute appendicitis. Ultrasonography has been reported to have a sensitivity of 90% and a specificity of over 90% in experienced hands [33]. Despite that, ultrasonography depends on the operator’s skill and may not always provide a clear view of the appendix due to increased bowel gases that localized bowel distension from inflammation causes. Therefore, a CT scan, when available, is considered to be more effective than ultrasonography for diagnosing acute appendicitis [33,34].

As for the treatment and clinical outcome, we found that conservative treatment was dominant, whereas about one-third of the cases underwent an open appendectomy. One-third of the cases with conservative treatment showed failure mainly within 45 days of the treatment. About half of the cases with failed conservative treatment had complicated appendicitis, with an average hospital stay of five days. Most high-risk cases underwent an open appendectomy, which is the trend globally. However, there has recently been advocacy for nonoperative care of uncomplicated appendicitis in some selected cases [35,36].

Limitation

The study was retrospective in nature, conducted in a single ER, and there may be missing data. Subsequently, every patient in the group with appendicitis complained of abdominal pain. Therefore, we did not find any patients who presented with appendicitis in atypical presentation.

## Conclusions

We found that most pediatric appendicitis cases were girls older than 10 years. In addition, all severity grades were reported almost equally among cases where more than half had simple appendicitis and one-third had complicated appendicitis. Wall thickness, enlarged appendix, or mass signs with signs of inflammation were the most reported in radiology. Conservative treatment was used for less than half of the cases with a 30% failure rate. The average duration of hospital stay among cases was less than one week.
